# Evaluation of Drug-Loading Ability of Poly(Lactic Acid)/Hydroxyapatite Core–Shell Particles

**DOI:** 10.3390/ma14081959

**Published:** 2021-04-14

**Authors:** Seiya Suzuki, Sungho Lee, Tatsuya Miyajima, Katsuya Kato, Ayae Sugawara-Narutaki, Makoto Sakurai, Fukue Nagata

**Affiliations:** 1 National Institute of Advanced Industrial Science and Technology (AIST), 2266-98 Anagahora, Shimoshidami, Moriyama-ku, Nagoya 463-8560, Japan; seiya-suzuki@aist.go.jp (S.S.); t.miyajima@aist.go.jp (T.M.); katsuya-kato@aist.go.jp (K.K.); 2 Department of Applied Chemistry, College of Engineering, Chubu University, Matsumoto-cho, Kasugai 487-8501, Japan; sakurai@isc.chubu.ac.jp; 3 Department of Energy Engineering, Graduate School of Engineering, Nagoya University, Furo-cho, Chikusa-ku, Nagoya 464-8603, Japan; ayae@energy.nagoya-u.ac.jp

**Keywords:** hydroxyapatite, poly(lactic acid), drug delivery system, loading capacity, core–shell particles

## Abstract

Poly(lactic acid)/hydroxyapatite (PLA/HAp) core–shell particles are prepared using the emulsification method. These particles are safe for living organisms because they are composed of biodegradable polymers and biocompatible ceramics. These particles are approximately 50–100 nm in size, and their hydrophobic substance loading can be controlled. Hence, PLA/HAp core–shell particles are expected to be used as drug delivery carriers for hydrophobic drugs. In this work, PLA/HAp core–shell particles with a loading of vitamin K_1_ were prepared, and their drug-loading ability was evaluated. The particles were 40–80 nm in diameter with a PLA core and a HAp shell. The particle size increased with an increase in the vitamin K_1_ loading. The drug-loading capacity (LC) value of the particles, an indicator of their drug-loading ability, was approximately 250%, which is higher than the previously reported values. The amount of vitamin K_1_ released from the particles increased as the pH of the soaking solution decreased because the HAp shell easily dissolved under the acidic conditions. The PLA/HAp particles prepared in this work were found to be promising candidates for drug delivery carriers because of their excellent drug-loading ability and pH sensitivity.

## 1. Introduction

Carriers that form part of drug delivery systems (DDS carriers) have attracted considerable attention [1]. DDS carriers are expected to deliver drugs to the appropriate sites in living organisms effectively and safely. In addition, the components of the DDS carriers should not remain in the body. Several materials, such as phospholipids, polyethylene glycol (PEG), and biodegradable polymers, have been used as DDS carriers. Phospholipids are liposomes composed of bilayer membranes [2]. The liposome is hydrophilic inside and has a hydrophobic membrane on the outside. PEG is a polymeric micelle composed of biocompatible molecules [3]. Polymeric micelles are formed from hydrophilic and hydrophobic polymers by the self-association of block copolymers. The degradation rate of microspheres with biodegradable polymers, such as poly(lactic acid) (PLA) and poly(lactic glycolic acid) (PLGA), can reportedly be controlled in vivo by adjusting their composition [4,5,6,7,8,9]. PLGA microspheres loaded with superparamagnetic iron oxide nanoparticles and dexamethasone acetate have been reported for the treatment of local inflammatory diseases [7]. PLGA microspheres/poly(vinyl alcohol) (PVA) hydrogel composites with dexamethasone and vascular endothelial growth factor have been reported to stimulate angiogenesis [8]. Inflamed areas exhibit greater vascular permeability and leakage of larger molecules than normal areas [10]; this is known as the enhanced permeability and retention effect (EPR effect) [11,12]. Nano-sized DDS carriers (20–200 nm) were designed to passively target inflammatory sites, which have interendothelial pores in the blood vessels [13]. Doxorubicin (DOX)-loaded liposomal preparations have been approved for the treatment of cancers such as Kaposi’s sarcoma using the EPR effect [14].

Hydroxyapatite (Ca_10_(PO_4_)_6_(OH)_2_ or HAp), a well-known bioceramic, is an inorganic component of bone. HAp exhibits excellent biocompatibility and bioresorbability [15]. Thus, various DDS carriers using HAp have been reported [16,17,18,19,20]. Tetracycline hydrochloride, an antibiotic, loaded onto porous HAp was composited with polycaprolactone, and the drug-release behavior of the composite was controlled by varying the mixing ratio [16]. Nanocomposites of DOX-loaded HAp and folic acid have shown enhanced drug effects on tumors [19].

Seventy percent of novel drugs are hydrophobic and the number of these drugs continues to increase [21]. Thus, the ability to load hydrophobic substances is a great advantage for DDS carriers. DDS carriers may reduce the burden on patients by decreasing the frequency of drug administration. However, carriers must have a large drug-loading capacity with a controlled release. Hence, improvements in the drug-loading capacity of DDS carriers have been studied [22,23]. Polymeric micelles have been developed to improve the drug-loading capacity via π–π stacking interactions [22] and by inducing additional π–π interactions [23]. The drug-loading capacity values of polymeric micelles with π–π stacking interactions and additional π–π interactions are 18% and 16%, respectively, which are larger than those of other poly(ε-caprolactone) and poly(*l*-lactide) polymer micelles (3–5%) [24]. Carbon dot particles can absorb drugs on their surfaces with a drug-loading capacity range of 310–439% [25]. Core–shell particles are normally composed of a core encapsulated within a shell, and the shell is expected to inhibit the reactivity and solubility of the materials loaded in the core. DOX-loaded poly(ethylene oxide)–poly(propylene oxide)–poly(ethylene oxide) block copolymers have been reported to be useful for multidrug-resistant cancer cells [26]. DOX-loaded Fe_3_O_4_/mesoporous nano-silica core–shell particles with a drug-loading capacity of 20% exhibit a suppression effect on the degradation and elution of DOX as compared to the administration of free DOX [27].

PLA particles for application as DDS carriers can be prepared using the emulsion method. However, it is necessary to use surfactants to stabilize the interface of these particles [28]. Moreover, a large amount of surfactants is required to prepare small particles [29]. Most surfactants are nonbiodegradable and tend to remain in the resulting particles [29]. In our previous work, we prepared PLA/HAp core–shell particles using the emulsification method without involving any surfactant [30,31,32]. The surface of PLA was stabilized with calcium ions, which bonded with the carboxyl groups in PLA [33]. Subsequently, HAp precipitated on the carboxyl groups, which bonded with the calcium ions; thus, the HAp shell was formed after aging for a certain duration [30,31]. Hydrophobic substances could be easily encapsulated within the PLA micelles of PLA/HAp core–shell particles. The particles were found to be safe for living organisms because they were composed of PLA and HAp. The particles were 40–100 nm in size and were expected to show the EPR effect on tumors and inflammatory sites. The present work represents a fundamental study of the drug-loading ability of PLA/HAp core–shell particles for application as DDS carriers. Vitamin K_1_ was chosen as the model drug for the evaluation of drug-loading capacity as it is a liquid fat-soluble vitamin. Vitamin K_1_-loaded PLA/HAp core–shell particles were prepared and their drug-loading capacity and drug-release behavior in phosphate buffer solutions were investigated.

## 2. Materials and Methods

### 2.1. Materials

Acetone (99.5%, Wako Pure Chemical Industries, Osaka, Japan), vitamin K_1_ (97%, Wako Pure Chemical Industries), Ca(CH_3_COO)_2_·H_2_O (99%, Wako Pure Chemical Industries), (NH_4_)_2_HPO_4_ (99%, Wako Pure Chemical Industries), PLA ((C_6_H_8_O_4_)_n_, Mw 10–18 kDa, Sigma-Aldrich, St. Louis, Missouri, United States), and ultrapure water (milli-Q, resistivity > 18.2 MΩ·cm) were used to prepare the PLA/HAp core–shell particles. A phosphate buffer solution was prepared using Na_2_HPO_4_ (99%, Wako Pure Chemical Industries) and KH_2_PO_4_ (99.5%, Wako Pure Chemical Industries) for drug-release testing.

### 2.2. Preparation of Vitamin K_1_-Loaded PLA/HAp Core–Shell Particles

**Scheme 1:**
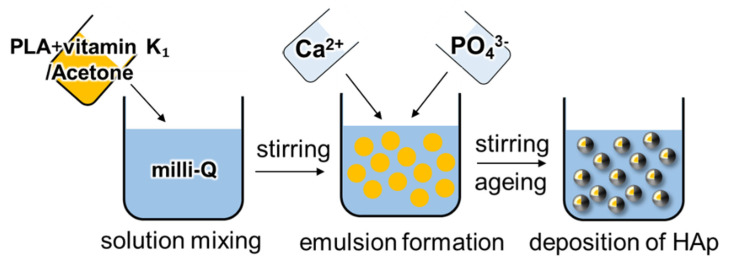
Preparation of the vitamin K_1_-loaded PLA/HAp core–shell particle solutions.

Vitamin K_1_-loaded PLA/HAp core–shell particles were prepared using an emulsification method (Scheme 1). The details of the preparation of the PLA/HAp core–shell particles are described in our previous paper [30,31,32]. In brief, PLA (20 mg) and various amounts of vitamin K_1_ (0, 50, 100, and 200 mg) were dissolved in 4 mL of acetone (denoted as PLHA-V*x*, where *x* is the amount of vitamin K_1_, (*x* = 0, 50, 100, 200)). This solution was then added to 160 mL of ultrapure water. To the resulting mixture, 20 mL of a 20 mM calcium acetate solution and 20 mL of a 12 mM potassium phosphate solution were added under stirring at 25 °C. The resulting solutions were aged for three days. The pH of the solutions was measured before and 1 h after the addition of the phosphate ions and after aging for 72 h (Table 1). The aged solutions were centrifuged (6000 rpm, 10 min). The supernatant was removed, and the particles were washed with ultrapure water. The particles were resuspended in 20 mL of ultrapure water. The residual vitamin K_1_ in the container was collected by dissolving it in ethanol, and its concentration was measured using ultraviolet–visible absorption spectroscopy (UV–Vis; JASCO Corporation, V-750) over the wavelength range of 200–900 nm (bandwidth: 2.0 nm; scanning speed: 400 nm/min). The absorption spectrum of vitamin K_1_ was set to 273 nm.

### 2.3. Characterization of the Vitamin K_1_-Loaded PLA/HAp Core–Shell Particles

The crystalline phase of PLHA-V*x* was evaluated using X-ray diffraction (XRD; Rigaku, Tokyo, Japan). The XRD conditions were as follows: CuKα radiation (40 kV, 30 mA); 1.0 °/min, and a 2θ range of 3–60°. PLHA-V*x* was evaluated by the attenuated total reflection (ATR) method using a Fourier transform infrared spectrometer (FT-IR; JASCO Corporation, Tokyo, Japan) in the range of 400–4000 cm^−1^. The morphology of PLHA-V*x* was observed using field emission scanning electron microscopy (FE-SEM; Hitachi, Tokyo, Japan). The PLHA-V*x*-containing solutions were dropped onto an aluminum sample stage and dried gently at 37 °C. Subsequently, PLHA-V*x* was coated with a layer of amorphous osmium (Meiwafosis, Tokyo, Japan). The diameters of the PLHA-V*x* particles were measured using a laser diffraction particle size analyzer (SALD-7500nano, Shimadzu, Kyoto, Japan). Particles weighing 5 mg were dispersed in 100 mL of ultrapure water and sonicated during the measurement. The PLHA-V0 solution was directly dropped onto a carbon grid and dried. The samples were observed using transmission electron microscopy (TEM; JEOL, Tokyo, Japan). The organic/inorganic ratio of PLHA-V*x* was evaluated using thermogravimetry–differential thermal analysis (TG–DTA; Rigaku, Tokyo, Japan). PLHA-V*x* (5 mg) was used for the analysis, and the same amount of Al_2_O_3_ was used as the reference. TG–DTA was performed at room temperature (approximately 25 °C) to 1000 °C (2 °C/min) under air flow (200 mL/min). For the XRD, FT-IR, and TG–DTA measurements, freeze-dried particles were used.

### 2.4. Drug-Release Test of the Vitamin K_1_-Loaded PLA/HAp Core–Shell Particles

PLHA-V200 was chosen for the drug-release test because it contained the highest amount of drug among all the PLHA-V*x* samples investigated. PLHA-V200 was added to phosphate buffer solutions with the pH values of 4.5, 5.5, and 7.4, and 1.88 mg of PLHA-V200 was soaked in 1 mL of the buffer solutions. The solutions were maintained at 37 °C for 1–6 days and stirred during the release test to prevent particle agglomeration. The phosphate buffer solutions (pH 4.5, 5.5, and 7.4) were prepared by mixing 66 mM Na_2_HPO_4_ and KH_2_PO_4_ in the Na_2_HPO_4_:KH_2_PO_4_ ratios of 0:20, 1:19, and 16:4 at pH = 4.5, 5.5, and 7.4, respectively. In this work, a protein-free phosphate buffer solution was selected for the drug-release test to investigate the fundamental properties of the PLA/HAp particles. The supernatant of the solution was collected by centrifugation (14,000 rpm) and subjected to Ultraviolet Visible Absorption Spectroscopy analysis (UV–Vis, JASCO Corporation, Tokyo, Japan).

## 3. Results

**Figure 1:**
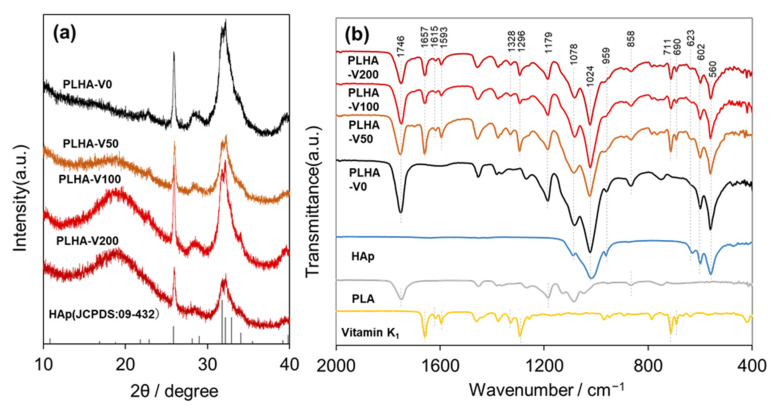
(**a**) X-ray diffraction (XRD) patterns and (**b**) Fourier transform infrared (FT-IR) spectra for the PLHA-V*x* particles.

During the particle preparation, the pH of the PLHA-V*x* solutions decreased after the addition of the phosphate ions (Table 1), indicating the precipitation of calcium phosphate. It is known that the pH decreases with precipitation of HAp [34]. The XRD patterns of PLHA-V*x* are shown in Figure 1a. PLHA-V*x* exhibited intense peaks at 2θ = 26° and 32°, corresponding to the 002 and 211 planes of HAp (JCPDS: 09-432), respectively. In addition, a halo peak at around 2θ = 18° was observed for PLHA-V50, -V100, and -V200. The FT-IR spectra of the particles are shown in Figure 1b. The bands corresponding to the phosphate group of HAp were the ν_2_ bending vibration at 469 cm^−1^; the ν_4_ bending vibration at 560, 602, and 623 cm^−1^; the ν_1_ symmetric stretching vibration at 959 cm^−1^; and the ν_3_ asymmetric stretching vibration at 1024 cm^−1^ [35,36,37]. The carboxyl group of PLA was assigned to the band at 858 cm^−1^ for the –C–COO stretching vibration, 1078 cm^−1^ for the COC symmetric stretching vibration, 1179 cm^−1^ for the COC asymmetric stretching vibration and CH_3_ asymmetric bending vibration, and 1746 cm^−1^ for the C=O stretching vibration [38]. In addition, the vitamin K_1_ bands of PLHA-V50, -V100, and -V200 were located at 1296 and 1328 cm^−1^ for the –C=C and C–C groups, 1374 cm^−1^ for the CH_3_ group, 1457 cm^−1^ for the CH_2_ group, 1593 and 1615 cm^−1^ for the C=C group, and 1657 cm^−1^ for the C=O group [39].

**Figure 2:**
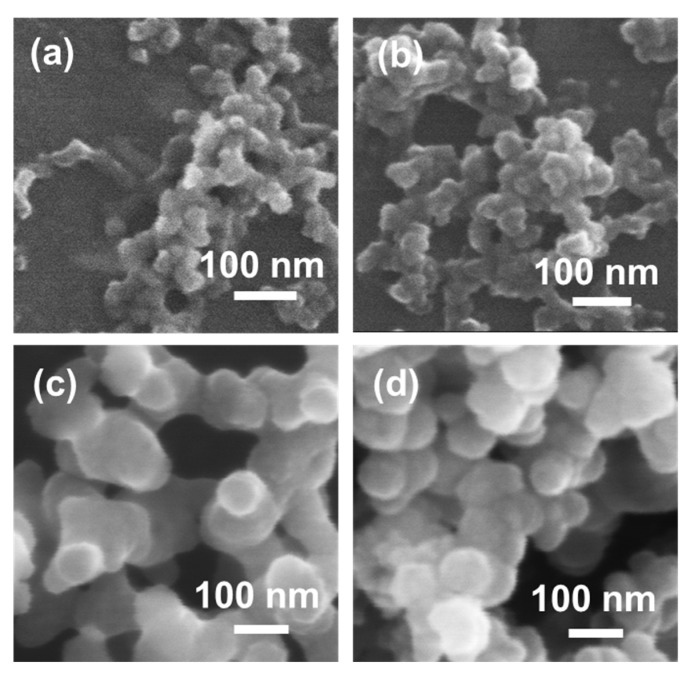
Scanning electron microscopy (SEM) images of (**a**) PLHA-V0, (**b**) PLHA-V50, (**c**) PLHA-V100, and (**d**) PLHA-V200.

**Figure 3:**
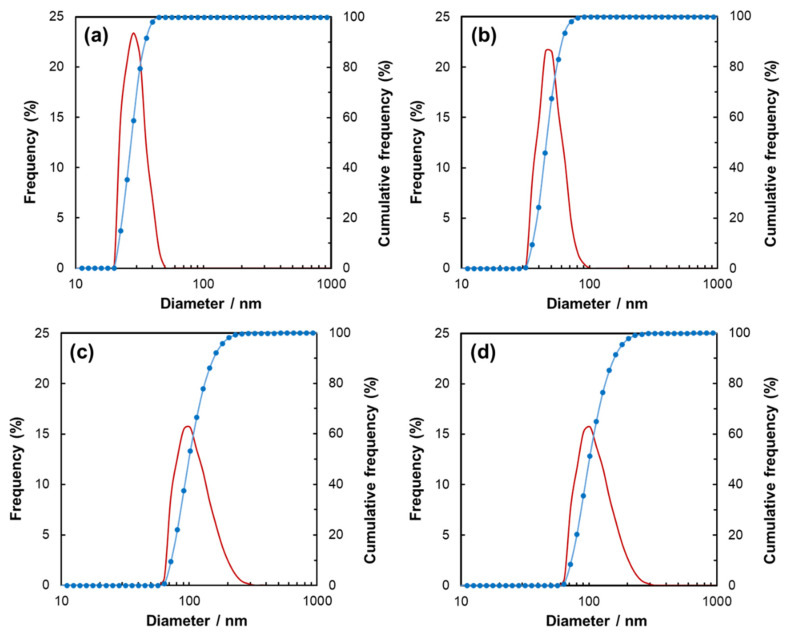
Particle diameter distribution of (**a**) PLHA-V0, (**b**) PLHA-V50, (**c**) PLHA-V100, and (**d**) PLHA-V200. Red solid lines represent the appearance frequency, and blue solid lines represent the cumulative frequency.

**Figure 4:**
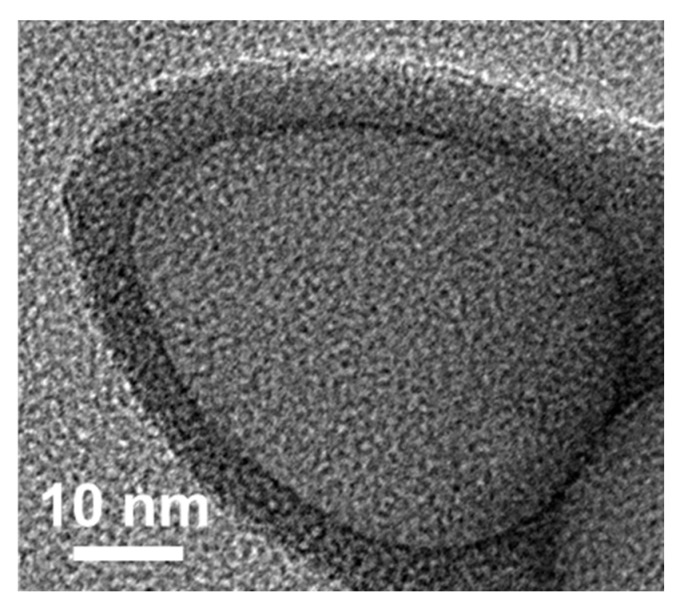
TEM image of PLHA-V0.

The PLHA-V*x* particles were spherical, as shown in the SEM images in Figure 2. The particle size distribution is shown in Figure 3. The average particle sizes of PLHA-V0, -V50, -V100, and -V200 were 27, 47, 104, and 105 nm, respectively. The particle size was increased with an increasing drug-loading amount. The TEM image of PLHA-V0 is shown in Figure 4. The inner gray sphere and black shell were approximately 40 nm in diameter and 6 nm in thickness, respectively. The percentages of the organic/inorganic components in PLHA-V*x* were estimated using TG–DTA (Table 1), where the inorganic component of PLHA-V*x* was HAp, and the organic component was the sum of PLA and vitamin K_1_. The amount of residual vitamin K_1_ in the container for the preparation of PLHA-V200 was measured to be 109 mg using UV–Vis spectroscopy. The loading amount of vitamin K_1_ in PLHA-V200 was calculated to be 91 mg.

**Figure 5:**
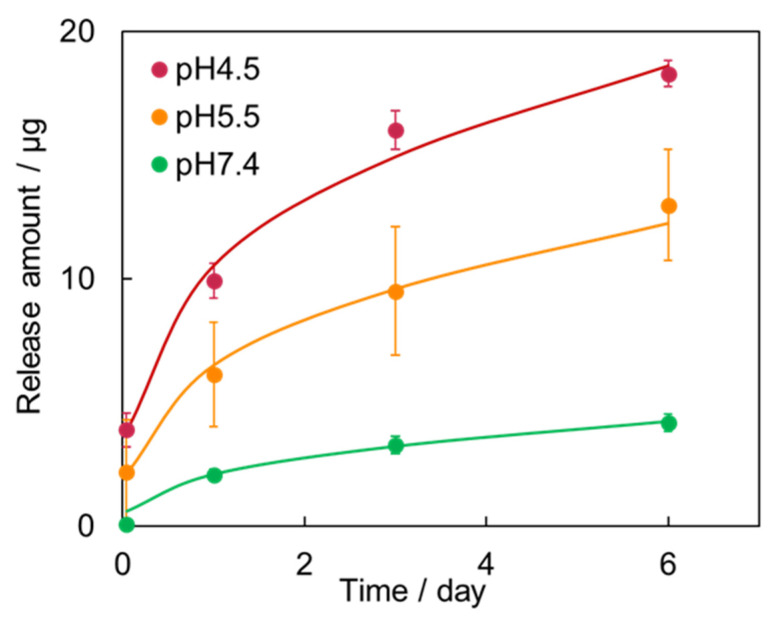
Vitamin K_1_-releasing behavior of PLHA-V200 in phosphate buffer at various pH levels. Error bars represent the standard deviation (*n* = 3). Solid lines represent Korsmeyer–Peppas fitted release profiles.

The vitamin K_1_-releasing behavior of PLHA-V200 in the phosphate buffer at various pH levels is shown in Figure 5. PLHA-V200 was chosen for the release test because it contained the largest amount of vitamin K_1_ among all the PLHA-V*x* samples investigated. Vitamin K_1_ was not found to undergo the initial burst release behavior in PLHA-V200 at all pH levels. The amount of vitamin K_1_ released in the phosphate buffer at pH 4.5, 5.6, and 7.4, was 18, 13, and 5 µg, respectively, and this amount increased as the pH of the phosphate buffer solution decreased. 

## 4. Discussion

The XRD patterns of the PLHA-V*x* particles showed peaks corresponding to HAp. PLHA-V50, -V100, and -V200 showed a halo peak centered at 2θ = 18°, which may have originated from vitamin K_1_. The intensity of this peak increased with an increase in the vitamin K_1_ content. This peak was confirmed for a mixture of PLHA-V0 and vitamin K_1_ (Figure S1 in Supplementary Materials). In addition, the XRD peaks of PLHA-V*x* were not sharp, which were similar to those of biological HAp [40,41]. The FT-IR spectra of PLHA-V*x* showed bands corresponding to the phosphate and carboxyl groups of HAp and PLA, respectively. The bands corresponding to vitamin K_1_ were observed in the spectra of PLHA-V50, -V100, and -V200, whereas these bands were not observed for PLHA-V0. The hierarchical architecture of PLHA-V0 was composed of a PLA core and a HAp shell, as shown in the TEM image. Additionally, the PLHA-V*x* particles were spherical in shape, as confirmed by the SEM images, and no other precipitation was visible. Thus, PLHA-V*x* was successfully prepared as a core–shell structure by loading vitamin K_1_.

The diameters of the PLHA-V0, -V50, -V100, and -V200 particles were 27, 47, 104, and 105 nm, respectively, and the size of particles increased with an increase in the amount of vitamin K_1_. Additionally, the organic component of PLHA-V*x* increased with the increasing amounts of vitamin K_1_. Hence, the particles expanded with an increase in the amount of drug that was encapsulated. 

The loading capacity (LC) of DDS carriers is an indicator of their drug-loading ability and can be calculated using the following equation [25,42,43]:

(1) $$\mathrm{LC}\;\left(\%\right)=\frac{W_{d}}{W_{c}}\times 100$$

where *W_d_* is the mass of the drug (mg) and *W_c_* is the mass of the DDS carriers (mg). In this work, the LC can be altered as follows.

(2) $$\mathrm{LC}\;\left(\%\right)=\frac{R_{V}}{R_{H}+R_{P}}\times 100$$

where *R_V_*, *R_H_*, and *R_P_* are the weight ratios of vitamin K_1_, HAp, and PLA in the particles, respectively. The *R_H_* and (*R_V_* + *R_P_*) ratios were obtained using TG–DTA (Table 2). In the case of PLHA-V200, the *R_V_* can be altered as follows:

(3) $$R_{V}=84.5-R_{p}$$

where *R_H_* is 15.5, as determined from the TG–DTA results. The weight ratio of *R_V_* and *R_P_* can be obtained as follows:

(4) $$R_{V}:R_{p}=W_{V}:W_{p}$$

where *W_V_* and *W_p_* are the weights of vitamin K_1_ and PLA (mg) in PLHA-V200, respectively. *W_P_* is the amount of PLA used for particle preparation, which was 20 mg. *W_V_* is the amount of vitamin K_1_ loaded into PLHA-V200, as obtained from the UV–Vis spectrum, which was 109 mg. *R_V_*:*R_P_* can be calculated as 71.4:13.1, from the values of *W_V_* and *W_P_* above. The weight ratio of HAp, PLA, and vitamin K_1_ in PLHA-V200 is 15.5, 13.1, and 71.4%, respectively. Consequently, the LC value of PLHA-V200 was calculated to be 250%. Zhang et al. reported that polymeric micelles with anticancer agents, which were prepared by exploiting the π–π interactions between biodegradable polymers and disulfide bonds, exhibited an LC value of 18% [22]. Li et al. reported that polymeric micelles with π–π conjugated moieties as lipophilic segments for delivering anticancer agents exhibited an LC value of 15% [23]. Al-Amin et al. reported liposome particles with an LC value of 12% [44]. Therefore, PLHA-V*x* exhibited an excellent drug-loading capacity, with an LC value of 250%.

The amount of vitamin K_1_ released from the particles increased as the pH of the phosphate buffer solution decreased. HAp is poorly soluble under neutral and basic conditions [45] and readily dissolves under acidic conditions. Thus, the HAp shell can easily dissolve at pH 4.5, and therefore, a larger amount of vitamin K_1_ was released compared with that at pH 7.4. Furthermore, PLHA-V*x* showed no initial burst release behavior, and vitamin K_1_ was released gradually during immersion. The release behavior of PLHA-V200 showed good correlation with the Korsmeyer–Peppas equation in pH 4.5, 5.5, and 7.4 (R^2^ > 0.95) [46]. These results suggest that PLA/HAp core–shell particles are excellent candidates for DDS carriers with a larger drug-loading capacity and pH sensitivity.

## 5. Conclusions

PLHA-V*x* was prepared, and its drug-loading ability was evaluated. PLHA-V*x* was composed of PLA and HAp and was spherical in shape with a diameter of 40–80 nm. The diameter of PLHA-V*x* increased with an increase in the amount of vitamin K_1_. Thus, vitamin K_1_ was successfully encapsulated in the PLA/HAp particles. The LC value of PLHA-V200 was 250%, which is larger than those reported previously. PLHA-V200 showed pH sensitivity and no initial burst release behavior. Thus, PLHA-V*x* is a potential candidate for DDS carriers because of its excellent drug-loading ability and pH sensitivity. 

## Figures and Tables

**Table 1:** pH values of the PLHA-V*x* solutions under different conditions.

Sample Code	Addition of Phosphate Ion		After Aging (72 h)
	Before	1 h after	
PLHA-V0	7.61	6.81	6.19
PLHA-V50	7.67	6.86	6.23
PLHA-V100	7.61	6.80	6.13
PLHA-V200	7.70	6.88	6.35

**Table 2:** Organic/inorganic percentage of PLHA-V*x*.

Sample	HAp (%)	Organic Component (%)
PLHA-V0	50.3	49.7
PLHA-V50	27.8	72.2
PLHA-V100	24.9	75.1
PLHA-V200	15.5	84.5

## Data Availability

Data sharing is not applicable to this article.

## Supplementary Materials

The following are available online at https://www.mdpi.com/article/10.3390/ma14081959/s1, Figure S1: XRD patterns of PLHA-V0 and PLHA-V0 + vitamin K_1_ mixture.
