# Supplementary material for: Evaluation of Drug-Loading Ability of Poly(Lactic Acid)/Hydroxyapatite Core–Shell Particles
Source: Materials (Basel). 2021 Apr 14;14(8):1959. doi: 10.3390/ma14081959 (PMC8070725; doi:10.3390/ma14081959)
Supplement: Supplementary file 1 [file materials-14-01959-s001.pdf]

materials

Supplementary materials

Evaluation of Drug-Loading Ability of Poly(Lactic Acid)/ Hydroxyapatite Core–shell Particles

Seiya Suzuki [1,2], Sungho Lee [1,*], Tatsuya Miyajima [1], Katsuya Kato [1], Ayae Sugawara-Narutaki [3], Makoto Sakurai [2] and Fukue Nagata [1,*]

[1] National Institute of Advanced Industrial Science and Technology (AIST), 2266-98 Anagahora, Shimoshidami, Moriyama-ku, Nagoya 463-8560, Japan; seiya-suzuki@aist.go.jp (S.S.); t.miyajima@aist.go.jp (T.M.); katsuya-kato@aist.go.jp (K.K.)
[2] Department of Applied Chemistry, College of Engineering, Chubu University, Matsumoto-cho, Kasugai, Aichi 487-8501, Japan; sakurai@isc.chubu.ac.jp
[3] Department of Energy Engineering, Graduate School of Engineering, Nagoya University, Furo-cho, Chikusa-ku, Nagoya 464-8603, Japan; ayae@energy.nagoya-u.ac.jp
* Correspondence: sungho.lee@aist.go.jp (S.L.); f.nagata@aist.go.jp (F.N.); Tel.: +81-52-736-7387 (F.N.)

Citation: Suzuki, S.; Lee, S.; Miyajima, T.; Kato, K.; Sugawara-Narutaki, A.; Sakurai, M.; Nagata, F. Evaluation of Drug-Loading Ability of Poly(Lactic Acid)/Hydroxyapatite Core–shell Particles. *Materials* **2021**, *14*, 1959. https://doi.org/10.3390/ma14081959

Academic Editor: Albena Lederer

Received: 26 February 2021
Accepted: 12 April 2021
Published: 14 April 2021

Publisher's Note: MDPI stays neutral with regard to jurisdictional claims in published maps and institutional affiliations.

Copyright: © 2021 by the authors. Licensee MDPI, Basel, Switzerland. This article is an open access article distributed under the terms and conditions of the Creative Commons Attribution (CC BY) license (http://creativecommons.org/licenses/by/4.0/).

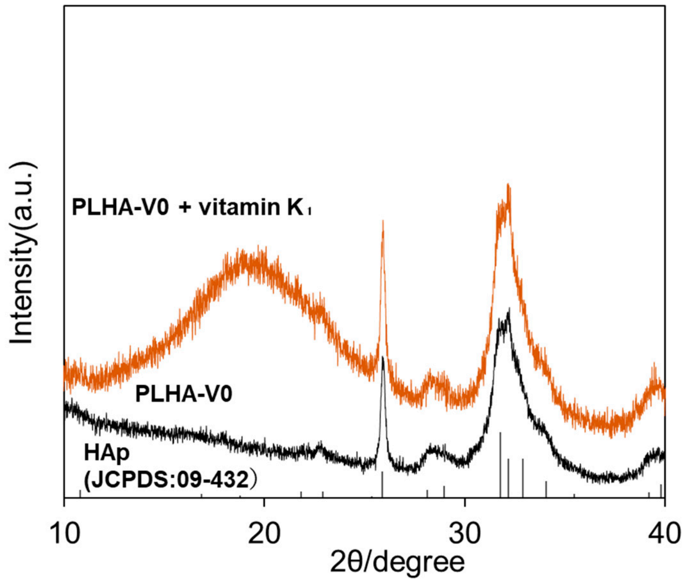

Figure S1. XRD patterns of PLHA-V0 and PLHA-V0 + vitamin K_1 mixture.

6 mg of PLHA-V0 and 3 mg of vitamin K_1 (liquid) was mixed for XRD measurement with non-reflective sample plate. The XRD conditions were as follows: CuKα radiation (40 kV, 30 mA), 2.0 deg/min, and a 2θ range of 3°–60° (XRD; Rigaku, SmartLab). A halo peak at 18° was observed in the mixture of PLHA-V0 and vitamin K_1. Thus, the halo peak originated from vitamin K_1 as an amorphous status.
